# Supplementary material for: Duplex sequencing provides detailed characterization of mutation frequencies and spectra in the bone marrow of MutaMouse males exposed to procarbazine hydrochloride
Source: Arch Toxicol. 2023 Jun 21;97(8):2245–59. doi: 10.1007/s00204-023-03527-y (PMC10322784; doi:10.1007/s00204-023-03527-y)
Supplement: Supplementary file 1 — Supplementary file1 (DOCX 1261 KB) [file 204_2023_3527_MOESM1_ESM.docx]

SUPPLEMENTARY TABLES & FIGURES

Supplementary Table 1. Mutagenesis panel targets and their chromosomal locations*

| **Target** | **Start Location** | **End Location** | **Genic Context** | **Chromatin State** | **%GC** |
| --- | --- | --- | --- | --- | --- |
| chr1 | 69304217 | 69306617 | Intergenic | Euchromatin | 37.3 |
| chr1.2 | 155235938 | 155238338 | Genic | Heterochromatin | 54 |
| chr2 | 50833175 | 50835575 | Intergenic | Heterochromatin | 45.3 |
| chr3 | 109633160 | 109635560 | Genic | Euchromatin | 39.2 |
| chr4 | 96825280 | 96827680 | Intergenic | Euchromatin | 39.4 |
| chr5 | 18210612 | 18213012 | Intergenic | Euchromatin | 35.6 |
| chr6 | 119170706 | 119173106 | Genic | Euchromatin | 40.5 |
| chr7 | 142683053 | 142685453 | Genic | Euchromatin | 45.9 |
| chr8 | 43954521 | 43956921 | Intergenic | Euchromatin | 35.6 |
| chr9 | 28648072 | 28650472 | Genic | Euchromatin | 37.3 |
| chr10 | 21442014 | 21444414 | Intergenic | Euchromatin | 46.3 |
| chr11 | 37934364 | 37936764 | Intergenic | Heterochromatin | 35.8 |
| chr12 | 80601542 | 80603942 | Genic | Heterochromatin | 52 |
| chr13 | 74030071 | 74032471 | Genic | Euchromatin | 50.4 |
| chr14 | 13076171 | 13078571 | Intergenic | Heterochromatin | 42.2 |
| chr15 | 66779762 | 66782162 | Genic | Euchromatin | 44 |
| chr16 | 72381580 | 72383980 | Intergenic | Heterochromatin | 38.3 |
| chr17 | 94009028 | 94011428 | Intergenic | Euchromatin | 35.2 |
| chr18 | 81262078 | 81264478 | Intergenic | Euchromatin | 47.3 |
| chr19 | 4618813 | 4621213 | Genic | Heterochromatin | 56.1 |

* mm10 coordinates; from LeBlanc et al., 2022.

Supplementary Table 2. Summary of DS mutation induction within the 24 MutaMouse bone marrow samples.

| **Sample** | **Dose** | **Mut Depth Min** | **Mut Depth Max** | **Total Depth** | **MF_Min_** | **MF_Max_** |
| --- | --- | --- | --- | --- | --- | --- |
| DNA02444 | 0 | 114 | 129 | 728699669 | 1.56443E-07 | 1.77028E-07 |
| DNA02445 | 0 | 122 | 135 | 817107673 | 1.50531E-07 | 1.65217E-07 |
| DNA02447 | 0 | 110 | 113 | 846953699 | 1.31058E-07 | 1.59395E-07 |
| DNA02448 | 0 | 87 | 96 | 780873955 | 1.11414E-07 | 1.4471E-07 |
| DNA02449 | 0 | 96 | 101 | 770727712 | 1.24558E-07 | 1.24558E-07 |
| DNA02450 | 0 | 94 | 99 | 826612593 | 1.13717E-07 | 1.22185E-07 |
| DNA02446 | 6.25 | 113 | 135 | 657402286 | 1.71889E-07 | 1.50593E-07 |
| DNA02451 | 6.25 | 138 | 189 | 820030039 | 1.68287E-07 | 2.30479E-07 |
| DNA02452 | 6.25 | 155 | 188 | 782634215 | 1.99327E-07 | 2.40214E-07 |
| DNA02453 | 6.25 | 108 | 141 | 853755000 | 1.265E-07 | 1.65153E-07 |
| DNA02454 | 6.25 | 146 | 181 | 855903938 | 1.71748E-07 | 2.11472E-07 |
| DNA02455 | 6.25 | 155 | 179 | 978513059 | 1.59426E-07 | 1.82931E-07 |
| DNA02456 | 12.5 | 169 | 213 | 763679775 | 2.22606E-07 | 2.78913E-07 |
| DNA02457 | 12.5 | 164 | 216 | 809078854 | 2.027E-07 | 2.6697E-07 |
| DNA02458 | 12.5 | 142 | 195 | 706111588 | 2.01101E-07 | 2.7616E-07 |
| DNA02459 | 12.5 | 177 | 238 | 891637902 | 1.98511E-07 | 2.66924E-07 |
| DNA02460 | 12.5 | 137 | 197 | 729478918 | 1.87805E-07 | 2.70056E-07 |
| DNA02461 | 12.5 | 152 | 233 | 750681115 | 2.03815E-07 | 3.10385E-07 |
| DNA02462 | 25 | 295 | 419 | 793981642 | 3.71545E-07 | 5.2772E-07 |
| DNA02463 | 25 | 275 | 431 | 841255951 | 3.26892E-07 | 5.12329E-07 |
| DNA02464 | 25 | 221 | 346 | 791686322 | 2.80414E-07 | 4.37042E-07 |
| DNA02465 | 25 | 243 | 374 | 756161273 | 3.22682E-07 | 4.94603E-07 |
| DNA02466 | 25 | 274 | 448 | 936111894 | 2.927E-07 | 4.78575E-07 |
| DNA02467 | 25 | 229 | 289 | 666280326 | 3.452E-07 | 4.33751E-07 |

**Supplementary Figure 1**. Correlation of the trinucleotide composition between the DS Mouse-50 Mutagenesis Panel and the mm10 *Mus musculus* autosomes. Data represents the proportion of trinucleotides.


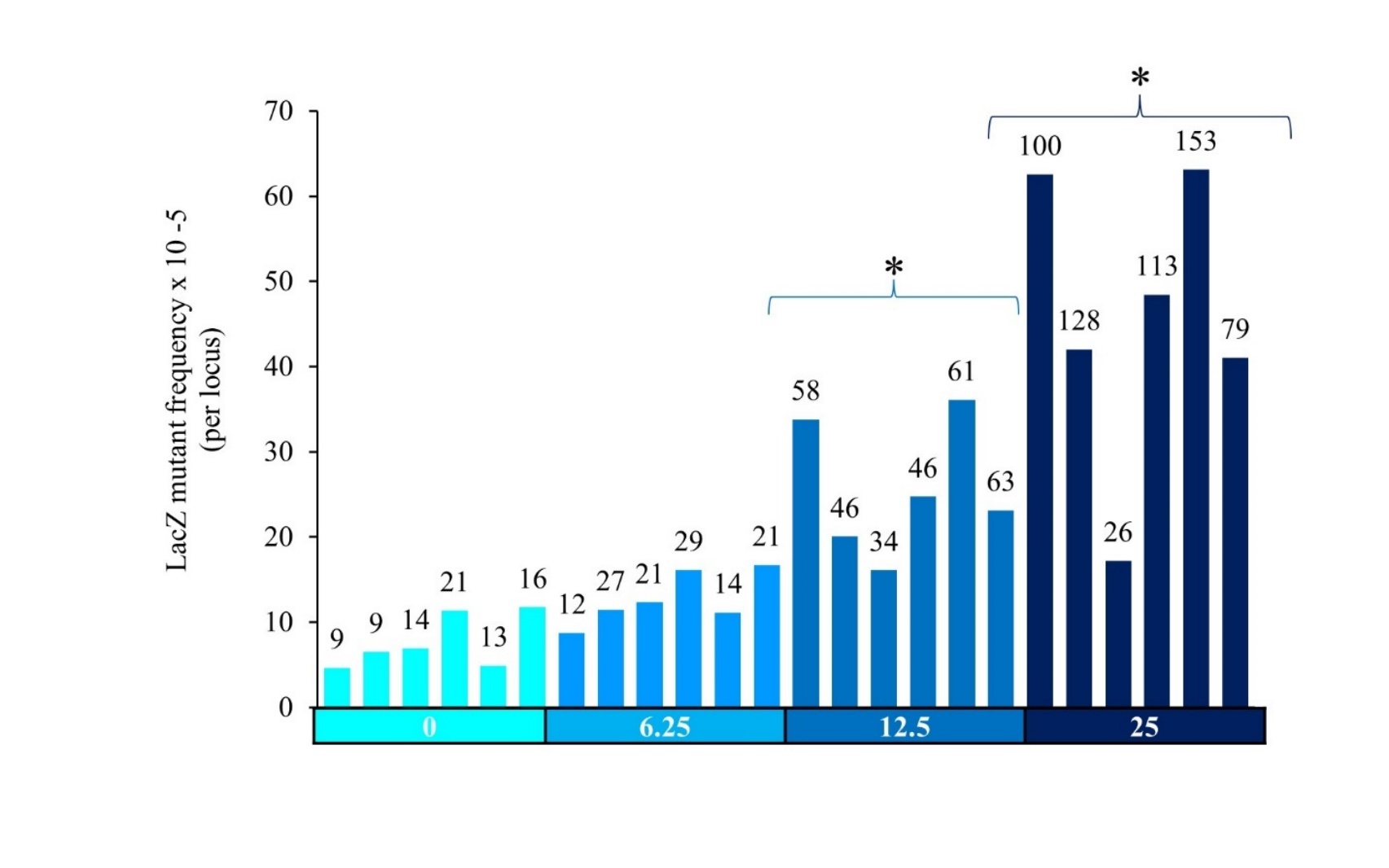
**Supplementary Figure 2**. *LacZ* mutant frequencies in the bone marrow of MutaMouse males at various doses of PRC. Bars represent mutant frequency (mutations per locus) for each animal. Data labels indicate total number of mutant plaques counted for each animal. The X-axis indicates PRC dose group (mg/kg-bw/day). Asterisks indicate a significant difference relative the controls in the average mutant frequency across animals,.A


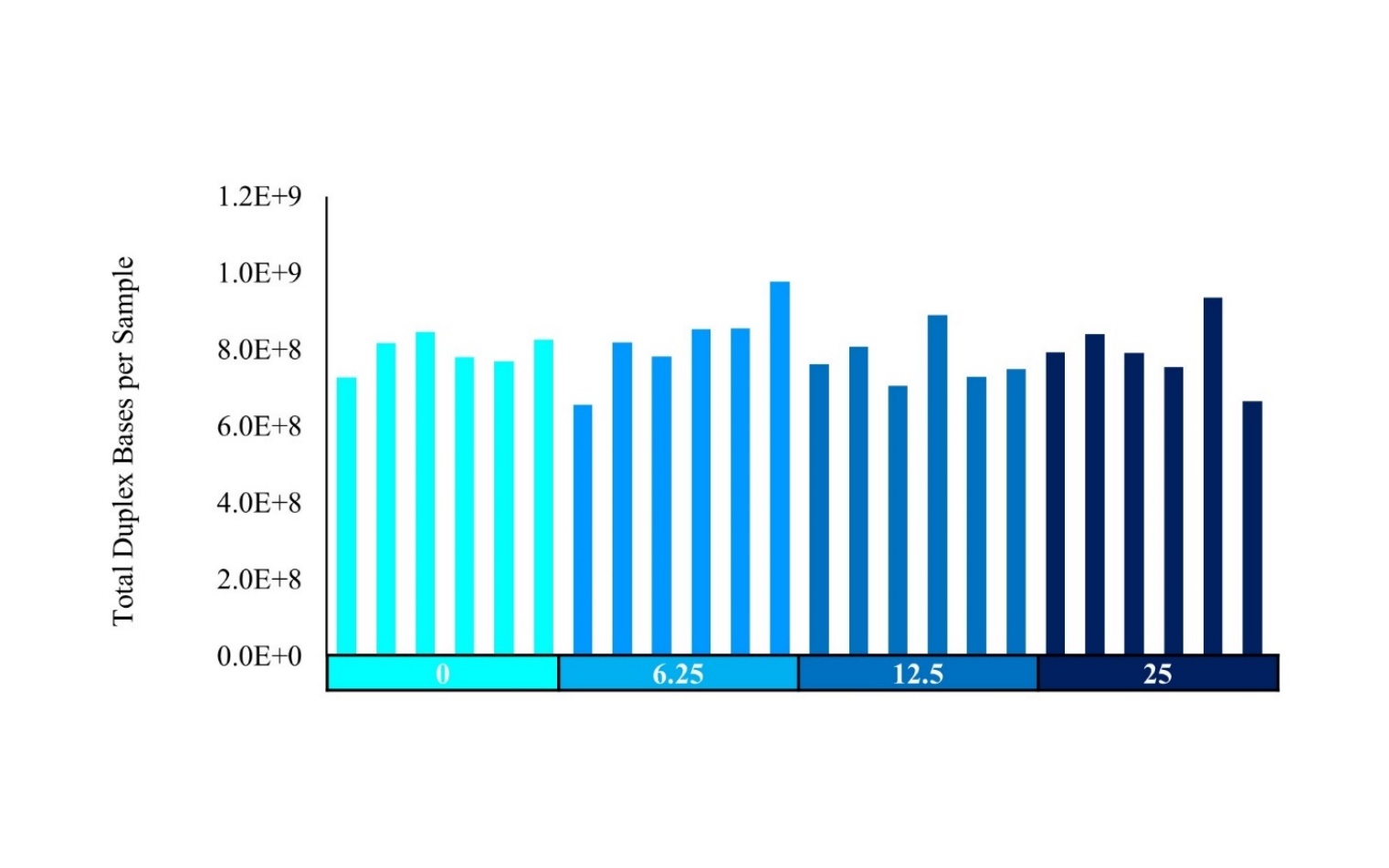


B
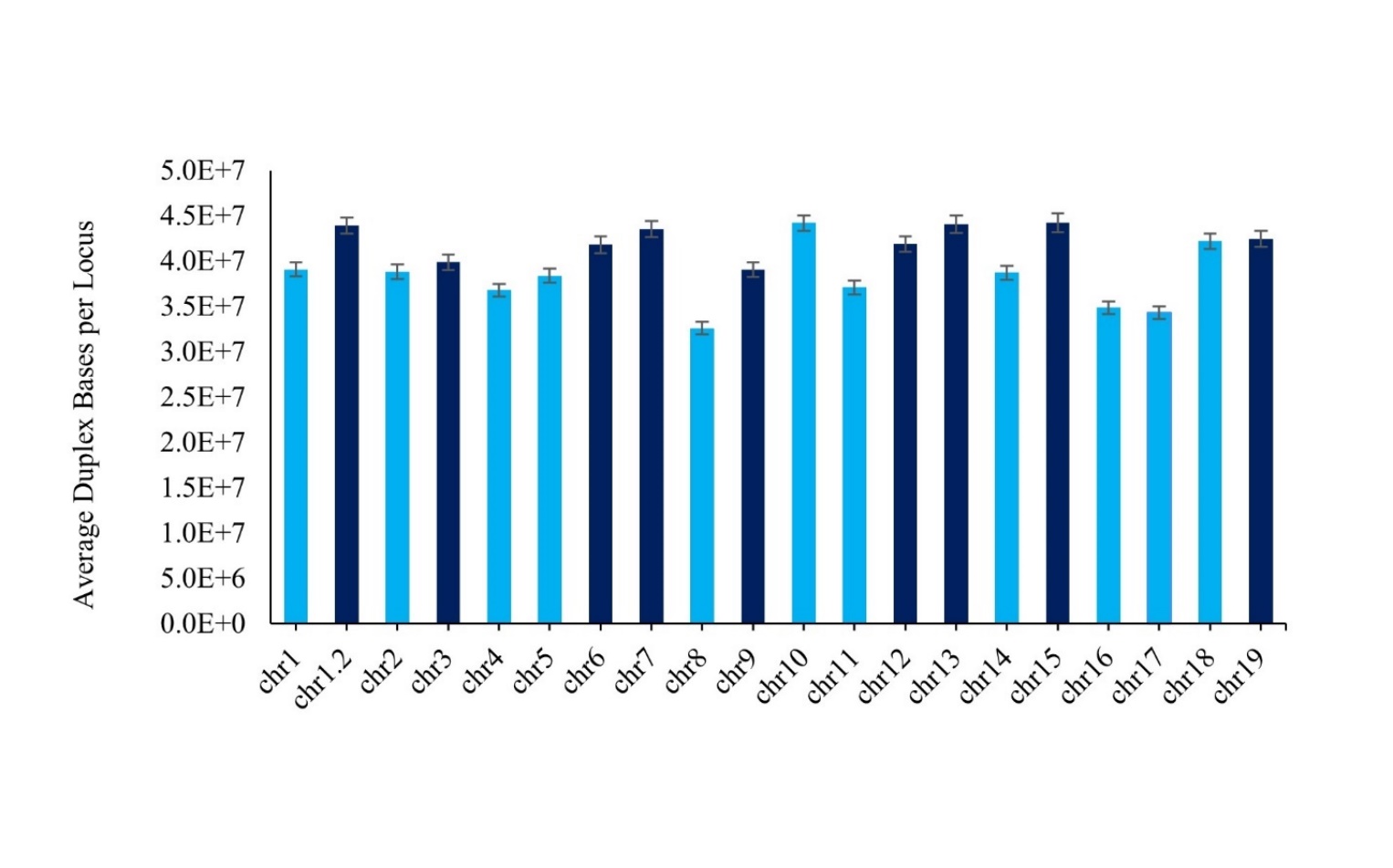
**Supplementary Figure 3**. Duplex data yields. (A) Total duplex bases per individual animal. X-axis indicates dose group (mg/kg-bw/day PRC). (B) Average duplex bases per chromosome location per animal. Error bars represent standard error of the mean (SEM).

A


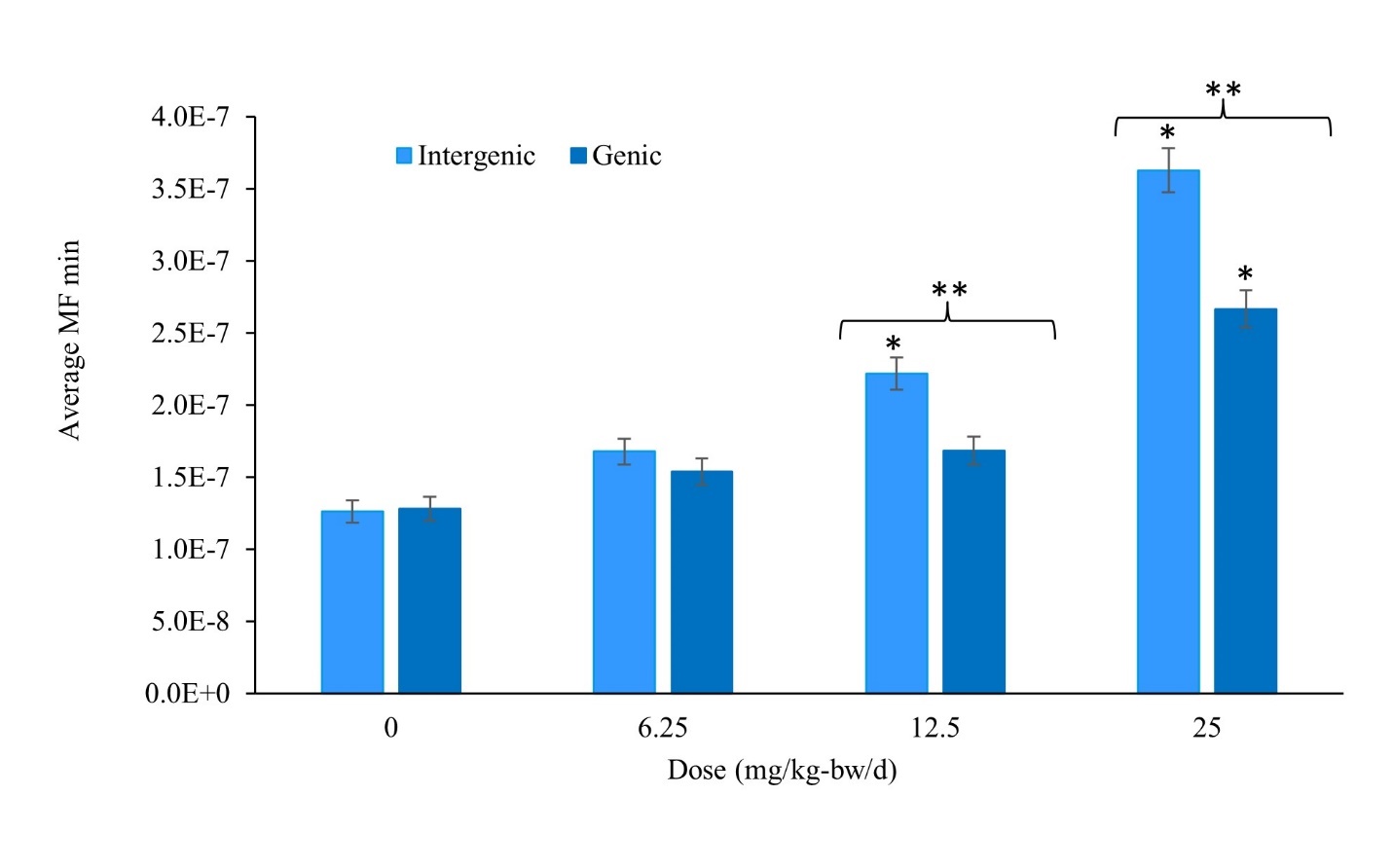


B


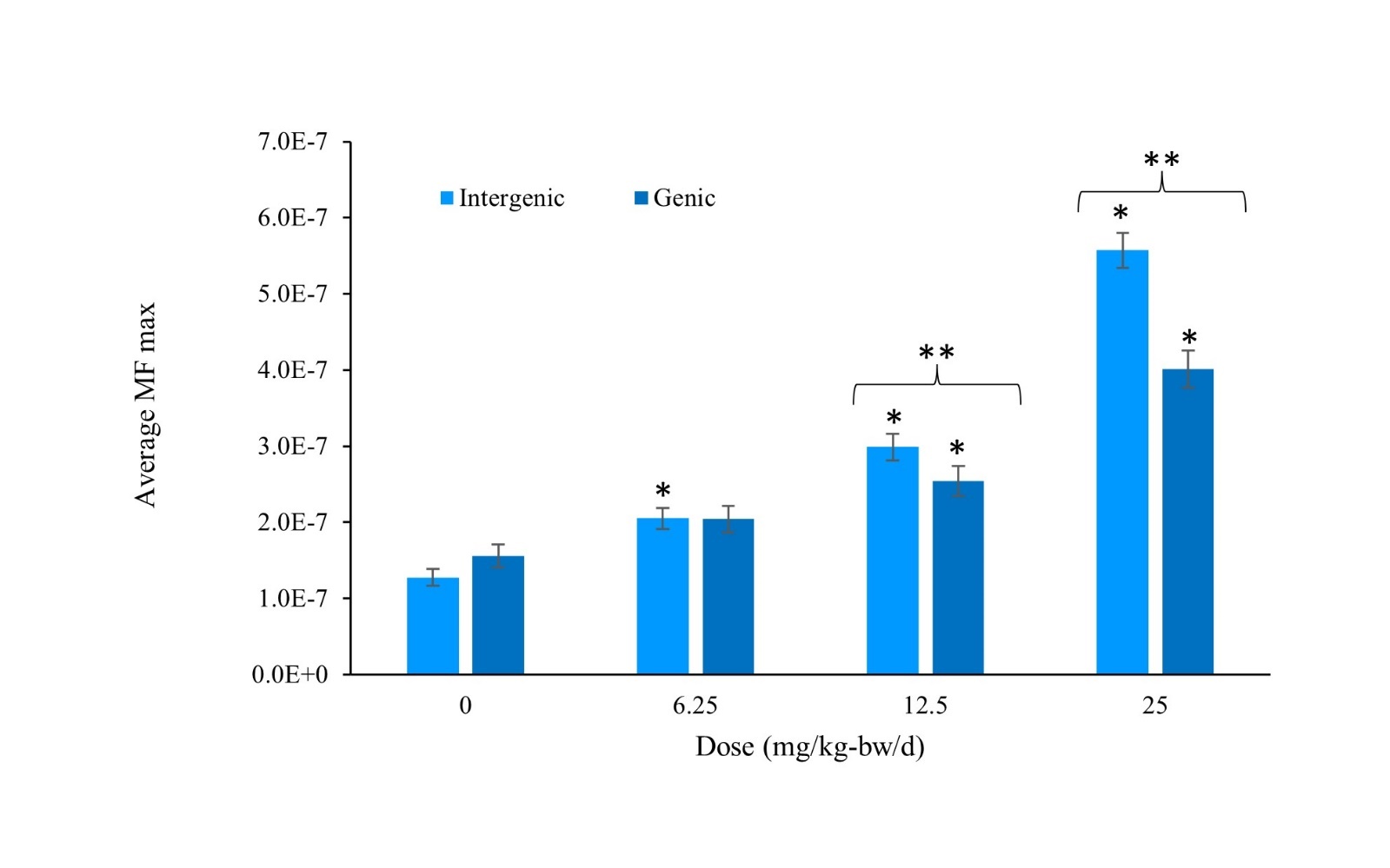


**Supplementary Figure 4**. MF of intergenic targets compared to genic targets for various doses of PRC using A) MF_Min_ and B) MF_Max_**.** Data are represented as the mean ± SEM (mutations per bp). Asterisks indicate a significant difference in MF of dose groups relative to controls (GLM, p < 0.05). Double asterisks indicate a significant difference in MF between intergenic and genic targets within a dose group (GLMM, p < 0.05).A
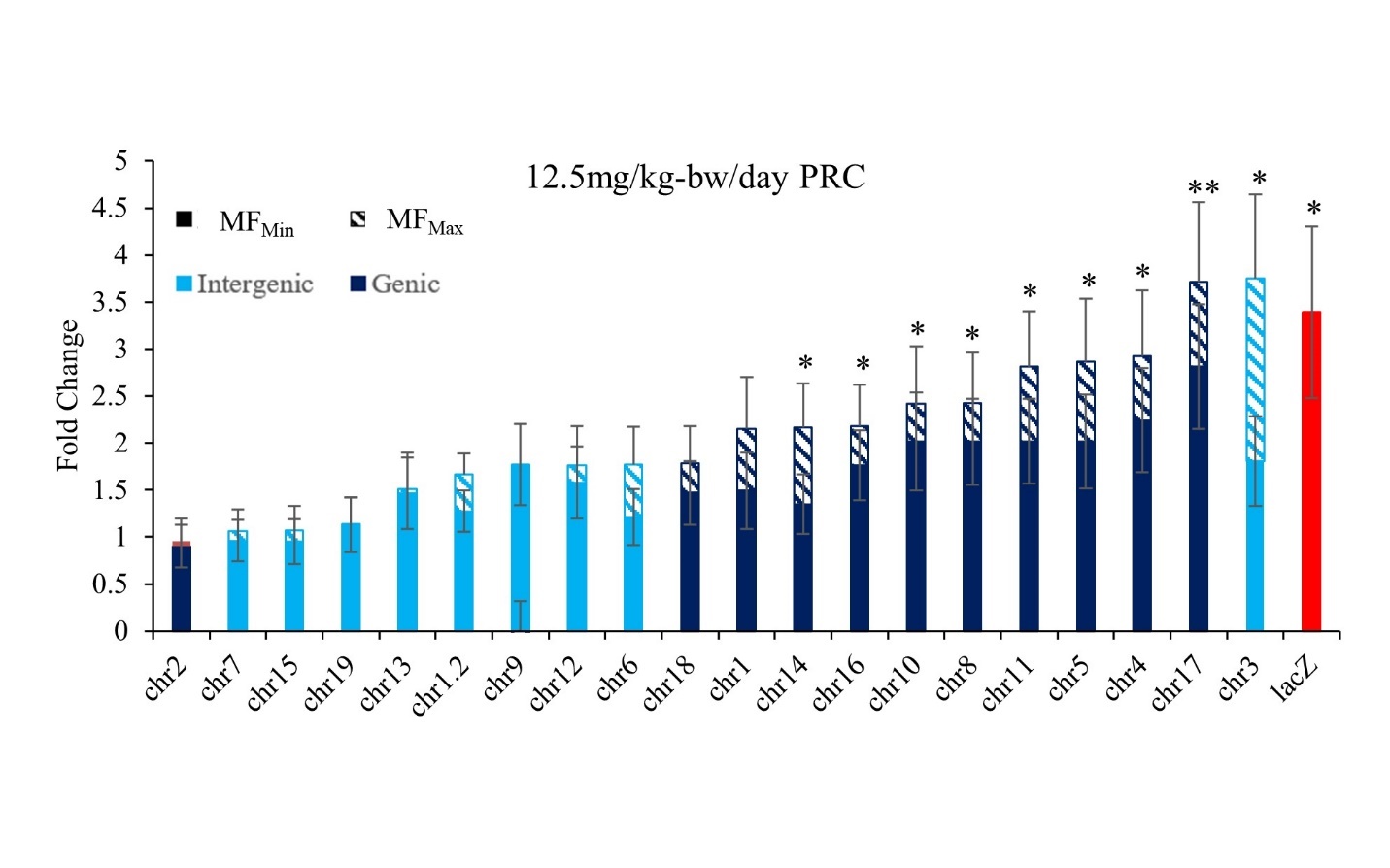
B
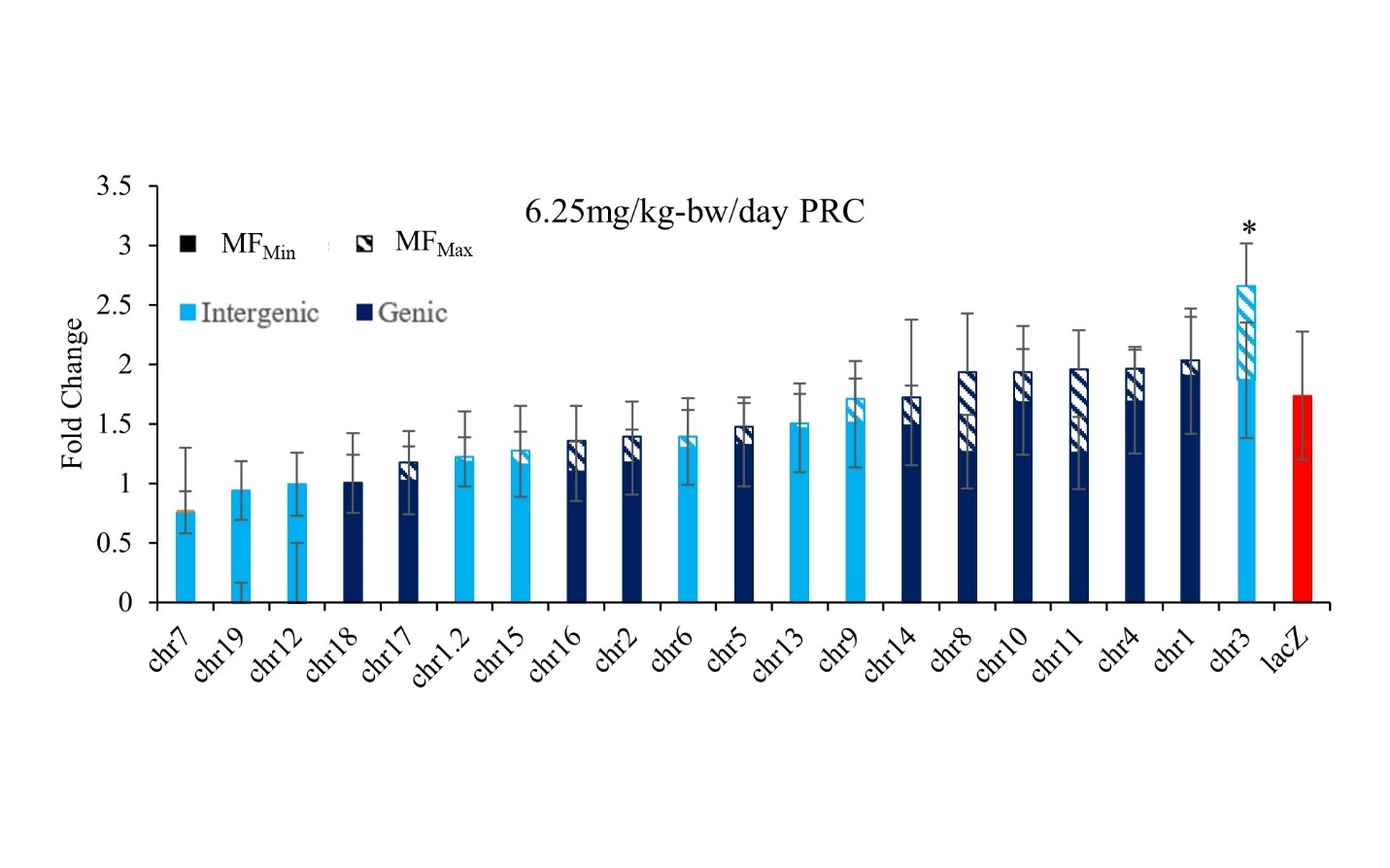
**Supplementary Figure 5.** Fold change in MF between PRC VC (**A**) and middle-dose group (**B**) and low-dose group for the 20 DS targets as well as for the *lacZ* gene (red bar), estimated using a general linear mixed model. DS targets are listed along the X-axis. Errors bars are SEM. Asterisks indicate the fold change was a significant increase in MF_Max_ from VC. Double asterisks indicate significant increase for both MF_Max_ and MF_Min_.

C>G

**
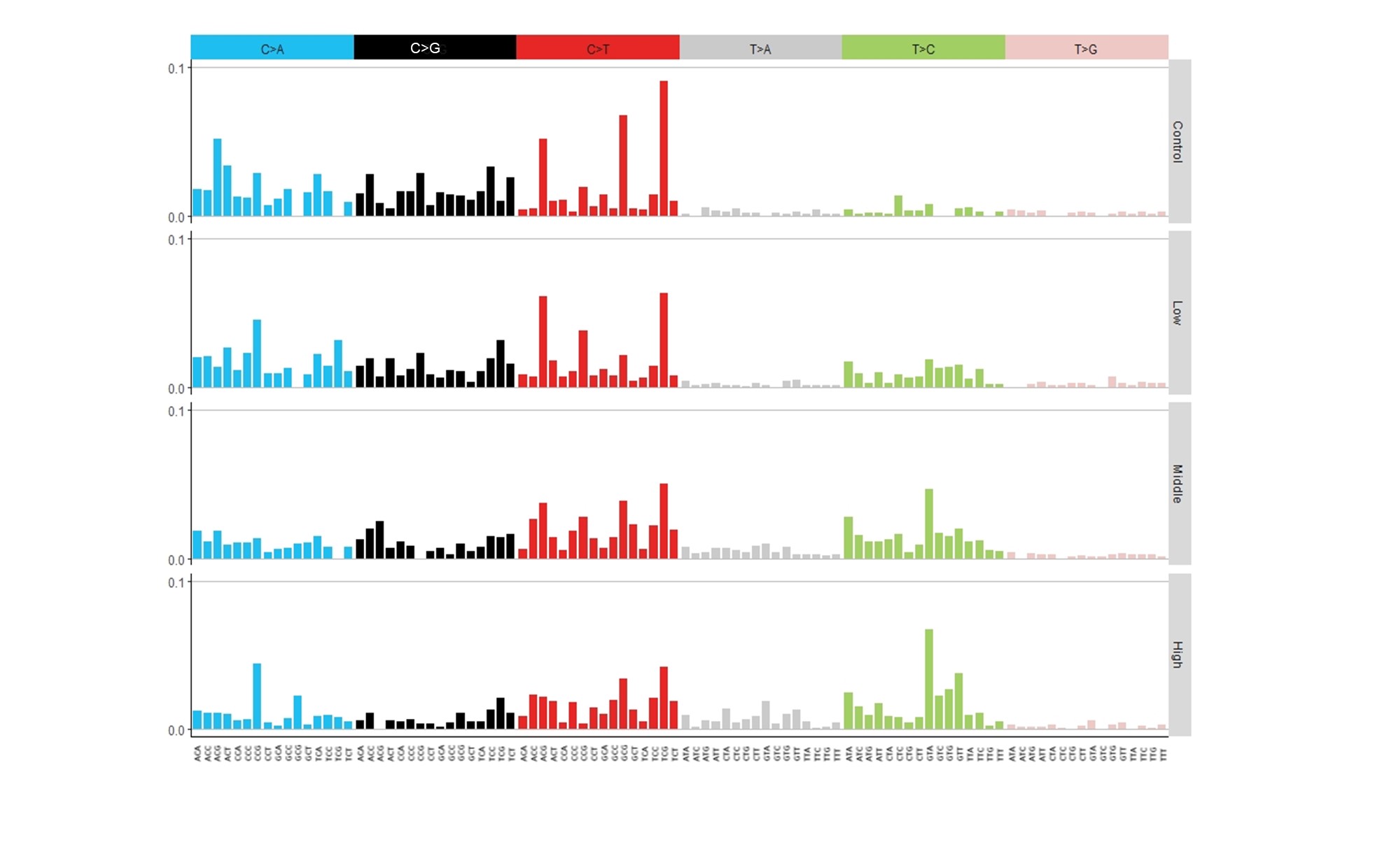
Supplementary Figure 6**. Mutation spectra with trinucleotide context of controls and PRC dose groups measured by Duplex Sequencing in the bone marrow of MutaMouse males. Dose groups are listed along the right: 6.25 mg/kg-bw/d (Low), 12.5 mg/kg-bw/d (Middle), 25 mg/kg-bw/d (High). The substitution subtype is listed along the top, the mutation including the two flanking nucleotides are listed along the bottom. Mutation subtypes are represented by the proportion of total mutations.**
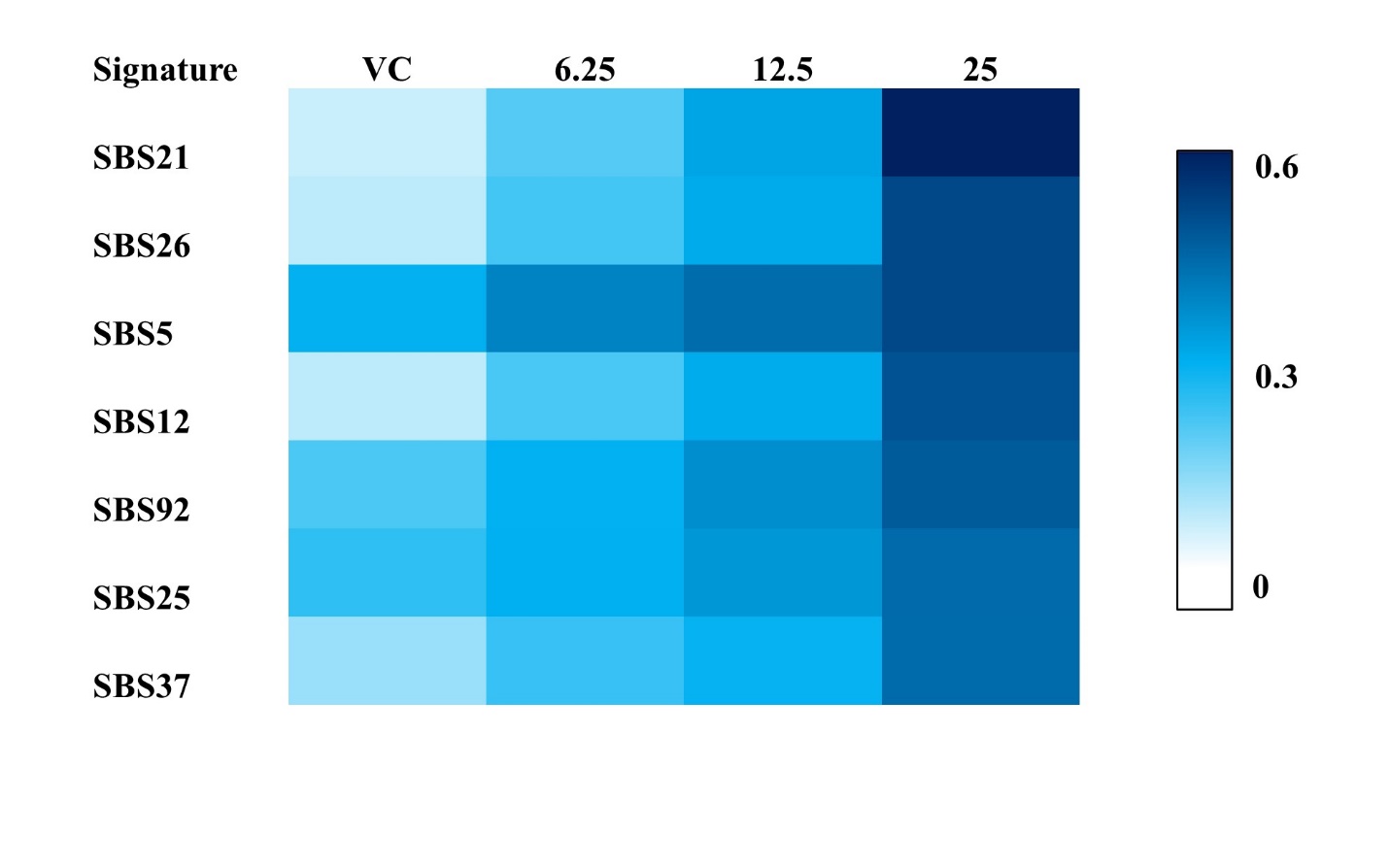
Supplementary Figure 7.** Cosine similarity of COSMIC SBS signatures to the trinucleotide mutation spectrum of each dose group. Data represents mean cosine value for the dose group.
